# How Healthy and Unhealthy Lifestyle Behaviors Affect Cognitive Function—Evidence From Older Adults in Chinese Communities: Cross-Sectional Study

**DOI:** 10.2196/73398

**Published:** 2025-08-12

**Authors:** Huixiu Hu, Yajie Zhao, Yuqing Hao, Huanhuan Luo, Lanying Xie, Chao Sun

**Affiliations:** 1 Department of Nursing, Beijing Hospital, National Center of Gerontology, Institute of Geriatric Medicine, Chinese Academy of Medical Science, No.1 Dahua, Dongcheng District, Beijing, 100730, China, 01085138594; 2 Department of Clinical Nutrition, Peking Union Medical College Hospital, Chinese Academy of Medical Science and Peking Union Medical College, Beijing, China; 3 Department of Cardiology, Beijing Hospital, National Center of Gerontology, Institute of Geriatric Medicine, Chinese Academy of Medical Science, Beijing, China; 4 Beijing Hospital, National Center of Gerontology, Institute of Geriatric Medicine, Chinese Academy of Medical Sciences and Peking Union Medical College, Beijing, China; 5 School of Nursing, Beijing University of Chinese Medicine, Beijing, China

**Keywords:** lifestyle behaviors, cognitive function, latent class analysis, health behavior promotion, behavioral synergy

## Abstract

**Background:**

Many lifestyle behaviors—including smoking, alcohol consumption, and engagement in physical activity and social activity—have been identified as potential determinants of the risk of cognitive impairment. Understanding how those lifestyle behavior patterns in older adults affect cognitive function is crucial for developing targeted interventions.

**Objective:**

This study examined the lifestyle behavior patterns of Chinese community-dwelling older adults and their associations with cognitive impairment.

**Methods:**

A cross-sectional study was conducted with 2060 community-dwelling older adults in Beijing, China. Latent class analysis identified distinct lifestyle behavior patterns based on unhealthy lifestyle behaviors (smoking and alcohol consumption) and healthy behaviors (physical activity and social activity). Cognitive function was evaluated using the Mini-Mental State Examination. Multiple logistic regression was conducted to examine the associations between lifestyle behavior patterns and cognitive impairment.

**Results:**

Three distinct lifestyle behavior patterns emerged: (1) high control-high engagement (685/2060, 33.3%), (2) high control-low engagement (1210/2060, 58.7%), and (3) low control-low engagement (165/2060, 8.0%). The high control-high engagement group, characterized by non-smoking, low-to-moderate alcohol consumption, and frequent engagement in physical and social activities, exhibited the lowest risk of cognitive impairment. In contrast, participants in the high control-low engagement group (OR 1.852, 95% CI 1.314-2.655) and low control-low engagement group (OR 2.905, 95% CI 1.670-5.001) exhibited significantly higher risks. Subgroup analyses revealed that males and hypertensive individuals within the high control-low engagement group were at an even greater risk.

**Conclusions:**

Our findings revealed that both avoiding harmful behaviors and actively engaging in health-promoting activities are important for cognitive health in older adults. Based on the results, we propose adopting a dual-pathway intervention model in policy making, simultaneously optimizing risk behaviors management and healthy behaviors promotion mechanisms.

## Introduction

With the global population aging, dementia has emerged as a critical public health challenge, with annual global costs exceeding USD 1.3 trillion [1]. China, in particular, is facing a mounting burden. Of the estimated 57.4 million individuals living with dementia worldwide in 2019, over one-quarter (15.3 million) resided in China [2]. Dementia-related deaths totaled 1.6 million globally that year, with China accounting for approximately 0.3 million deaths (19.8% of the global total) [3]. This demographic trend poses significant threats to the sustainability of health care systems unless cost-effective preventive strategies are implemented.

Given the limitations of pharmacological treatments, including high costs, potential side effects, and uncertain long-term safety and efficacy, identifying modifiable risk factors and implementing early interventions are critical for dementia prevention. The Lancet Commission [4] emphasized that up to 40% of dementia cases could be delayed or prevented by addressing modifiable risk factors through lifestyle interventions. Among the most modifiable are physical activity, social engagement, alcohol consumption, and smoking, each of which has well-established intervention frameworks. Evidence suggests that behavioral modifications targeting these domains not only enhance cognitive function in older adults but also reduce dementia incidence [5-8].

The evidence for cognitive health benefits of changing individual healthy lifestyle behaviors is robust. Many lifestyle behaviors—including smoking, alcohol consumption, and engagement in physical activity and social activity—have been identified as potential determinants of the risk of cognitive impairment. Moreover, there is some evidence that healthy behaviors often co-occur or cluster [9]. Existing studies frequently adopt a “one-size-fits-all” approach by aggregating multiple lifestyle factors into a single score [10-12]. Such an approach may obscure meaningful subgroup heterogeneity, limiting the precision of policy and intervention strategies [13]. Latent class analysis offers an alternative person-centered approach that classifies individuals into distinct classes based on shared characteristics, even when they appear similar in composite scores [14]. By maximizing between-group variation while minimizing within-group differences, LCA enables more nuanced insights into complex behavior-cognition relationships and supports the identification of distinct risk profiles in diverse older adults.

Recently, several studies have used latent class or profile analysis to identify health lifestyle-related behavior profiles and investigate the association with cognitive function [15-22]. Among these, 5 studies have focused exclusively on healthy lifestyle behaviors. However, simultaneously examining both health-promoting and health-risk behaviors allows for a more comprehensive and ecologically valid understanding of the full lifestyle continuum [15-20]. Notably, Norton et al [21] and Dingle et al [22] investigated both healthy and unhealthy behaviors among American and Australian adults, respectively. Importantly, the majority of existing evidence is derived from Western populations, whose lifestyle norms differ substantially from those in collectivist societies such as China [23]. For instance, in China, behaviors such as alcohol consumption and participation in physical activities are often embedded within social contexts that diverge markedly from those in Western cultures. As such, the generalizability of prior findings to the Chinese population remains uncertain and warrants further investigation.

In this study, we performed LCA to derive distinct lifestyle behavior classes based on both unhealthy lifestyle behaviors (smoking and alcohol consumption) and healthy behaviors (physical activity and social activity). We then examined the associations between these classes and the risk of cognitive impairment. Our findings may support the identification of older adult subgroups that would benefit most from targeted behavioral interventions aimed at reducing cognitive decline.

## Methods

### Study Design and Participants

A cross-sectional study was conducted from January to June 2023 in the 2 communities located in different districts of Beijing, China. Participants were recruited through community health service centers, which serve as primary care hubs in Chinese neighborhoods. Eligible older adults were approached by trained research assistants during routine medical visits or physical examinations. After receiving a detailed explanation of the study’s aims and procedures, individuals who provided informed consent were enrolled. Additional recruitment strategies included referrals by health care providers and digital outreach through WeChat-based community groups. All participants were screened and enrolled based on predefined inclusion and exclusion criteria. For older adults, the inclusion criteria were (1) aged 60 years or older and (2) having lived in this community for 6 months or longer. The residents were excluded if they had (1) vision or hearing impairment, preventing the completion of data collection and (2) mental disorders, such as schizophrenia and depression.

### Measurement Indicators

#### Lifestyle Behaviors

Based on a previous study [12], this study focused on unhealthy lifestyle behaviors (smoking and alcohol consumption) and healthy behaviors (physical activity and social activity). Each behavior was dichotomized and scored as either healthy (score=1) or unhealthy (score=0). Smoking was categorized into a current smoker (smoking) and not a current smoker (non-smoking). The score for non-smoking status was regarded as 1; otherwise, 0. Alcohol consumption was categorized into no-to-moderate consumption (up to 1.5 standard drinks per day) or heavy consumption. The score of no-to-moderate consumption was regarded as 1; otherwise, it was 0. Physical activity was classified into 2 categories: regular engagement in physical activity, defined as engaging in physical activity for at least 3 days per week with each session lasting ≥30 minutes, and less frequent physical activity. Examples of physical activities included brisk walking, jogging, Tai Chi, dancing, and aerobics. Regular physical activity was assigned a score of 1, while less frequent physical activity was scored 0. The score of weekly physical activity was regarded as 1; otherwise, it was 0. Finally, social engagement was categorized as active social engagement (participating in social activities at least 3 days per week (score=1) versus less frequent or no social activities (score=0). Social activities encompassed interacting with friends, playing mahjong, chess, or cards, visiting a community club, and attending sports events.

#### Cognitive Function

Cognitive function was assessed using the Chinese version of the Mini-Mental State Examination (MMSE). The MMSE evaluates 5 domains: orientation, memory, attention and calculation, recall, and language. The total score ranges from 0 to 30, with higher scores indicating better cognitive function. Cutoff points for cognitive impairment were determined based on education level, according to the latest normative and validation studies of the MMSE in the Chinese population. Cognitive impairment was defined as a score of ≤17 for individuals with no formal education, ≤20 for those with 1-6 years of education, and ≤24 for those with more than 6 years of education [24].

#### Covariates

Covariates were selected on the basis of previous evidence of associations with lifestyle and cognitive function and were ascertained by self-report. Sociodemographic covariates included gender (male or female), age in years, and marital status (married; or single, divorced, or widowed). Socioeconomic covariates included years of schooling (≤6 years, 7‐9 years, 10‐12 years, >12 years of education) and family economic status (poor, average, and rich). Chronic conditions were ascertained based on self-report of clinical diagnosis and included coronary heart disease, hypertension, diabetes, and hyperlipidemia. Self-report of clinical diagnosis of chronic conditions has been shown to have good agreement with ascertainment based on medical records [25]. The health status covariates included BMI (underweight, normal weight, overweight, and obesity) and annual physical examination (yes or no).

### Data Collection and Quality Control

Registered nurses in community health service centers who had more than 1 year of experience were considered research assistants. Unified training in administering the questionnaire and data collection was provided to the research assistants by researchers. Questionnaires were collected in the community health center. Researchers obtained written informed consent from the patients willing to participate and told them they could withdraw from the study at any point. Well-trained research assistants collected the information through face-to-face, one-on-one interviews.

### Data Analysis

Initially, LCA was performed to identify lifestyle behavior classes at baseline. Starting with a 1-category model, we systematically increased the number of classes, determining the optimal model by considering model fitting indices and the real-world significance of each category. The model fitting indices comprised (1) Akaike information criteria (AIC), Bayesian information criteria (BIC), and adjusted Bayesian information criteria, assessing model fit, with lower values indicating better fit; (2) entropy, reflecting the model’s classification quality, ranging from 0 to 1, with values closer to 1 indicating higher precision; and (3) likelihood ratio test and bootstrapped likelihood ratio test for comparing consecutive models, where a *P* value less than .05 indicated the Kth model had a better fit than the K–1th model.

To verify the relationship between lifestyle behavior classes and the risk of cognitive impairment, we used binary logistic regression analysis. The outcome variable of interest was the occurrence of cognitive impairment, with the independent variable being the lifestyle behavior classes. We performed a univariate analysis, followed by a comprehensive multivariate analysis that adjusted for all relevant covariates. Subsequently, we performed binary logistic regression analyses within each covariate-defined subgroup to investigate the association between health behavior profiles and the risk of cognitive impairment specific to those subgroups. Furthermore, we examined the joint effects of individual covariates and health behavior profiles on the likelihood of cognitive impairment, providing a nuanced understanding of the variables’ combined impact.

LCA was conducted in Mplus version 8.3 (Muthen & Muthen). The other statistical analyses were conducted using R (version 4.4.0; R Foundation for Statistical Computing), with binary logistic regression analysis leveraging the glm function. Moreover, additional visualizations were created using the versatile ggplot2 package. All cases with missing values have been deleted. The significance level for the 2-tailed test was established at *P* less than .05.

### Ethical Considerations

Ethics approval for this study was obtained from the Ethics Committee of Beijing Hospital (approval number: 2023BJYYEC-446‐02). Written informed consent was obtained from all participants prior to their participation. All the study data are anonymous. None of the participants received compensation. No images in the manuscript or supplementary materials contain any identifiable information of participants.

## Results

### Baseline Characteristics

The study screened 2434 older adults, ultimately including 2060 participants. Participants ranged in age from 60 to 94, with a mean age of 70.86 (SD 6.40). The 1203 women made up 58.4% of the population, with 857 men making up the remaining 41.6%. The 228 (11.0%) older adults had cognitive scores suggesting cognitive impairment, with an MMSE score of 28.44 (SD 2.59). The detailed results are presented in Table 1.

**Table 1.** General information of included community-dwelling older adults (n=2060).

Variable	n (%)
Gender	
Male	857 (41.6)
Female	1203 (58.4)
Age (years)	
<75	1534 (74.5)
≥75	526 (25.5)
Marital status	
Married	1938 (94.1)
Single, divorced, or widowed	122 (5.9)
Years of schooling (years)	
≤6	140 (6.8)
7‐9	448 (21.7)
10‐12	755 (36.7)
>12	717 (34.8)
Family economic status	
Poor	146 (7.1)
General	1337 (64.9)
Rich	577 (28.0)
Coronary heart disease	
No	1469 (71.3)
Yes	591 (28.7)
Hypertension	
No	697 (33.8)
Yes	1363 (66.2)
Diabetes	
No	1347 (65.4)
Yes	713 (34.6)
Hyperlipidemia	
No	1805 (87.6)
Yes	255 (12.4)
BMI	
Underweight	29 (1.4)
Normal	914 (44.4)
Overweight	857 (41.6)
Obesity	260 (12.6)
Annual physical examination	
No	359 (17.4)
Yes	1701 (82.6)

### Latent Class Analysis of Lifestyle Behaviors

We derived 4 models via the LCA, differing in terms of the number of lifestyle behaviors classes. Table 2 presents the fit indices for the latent classes. The Akaike information criteria, Bayesian information criteria, and adjusted Bayesian information criteria values kept decreasing from the first to the third model. The LMR and bootstrapped likelihood ratio test remained significant (*P*<.05) up to the 3-class model and then became non-significant. The entropy value was the highest in the 2-class model. Moreover, the conceptual sense of the 3-class model in the clinic was more significant than the 2-class model. Taking into account results obtained, we chose the solution with 3 classes.

**Figure 1.**
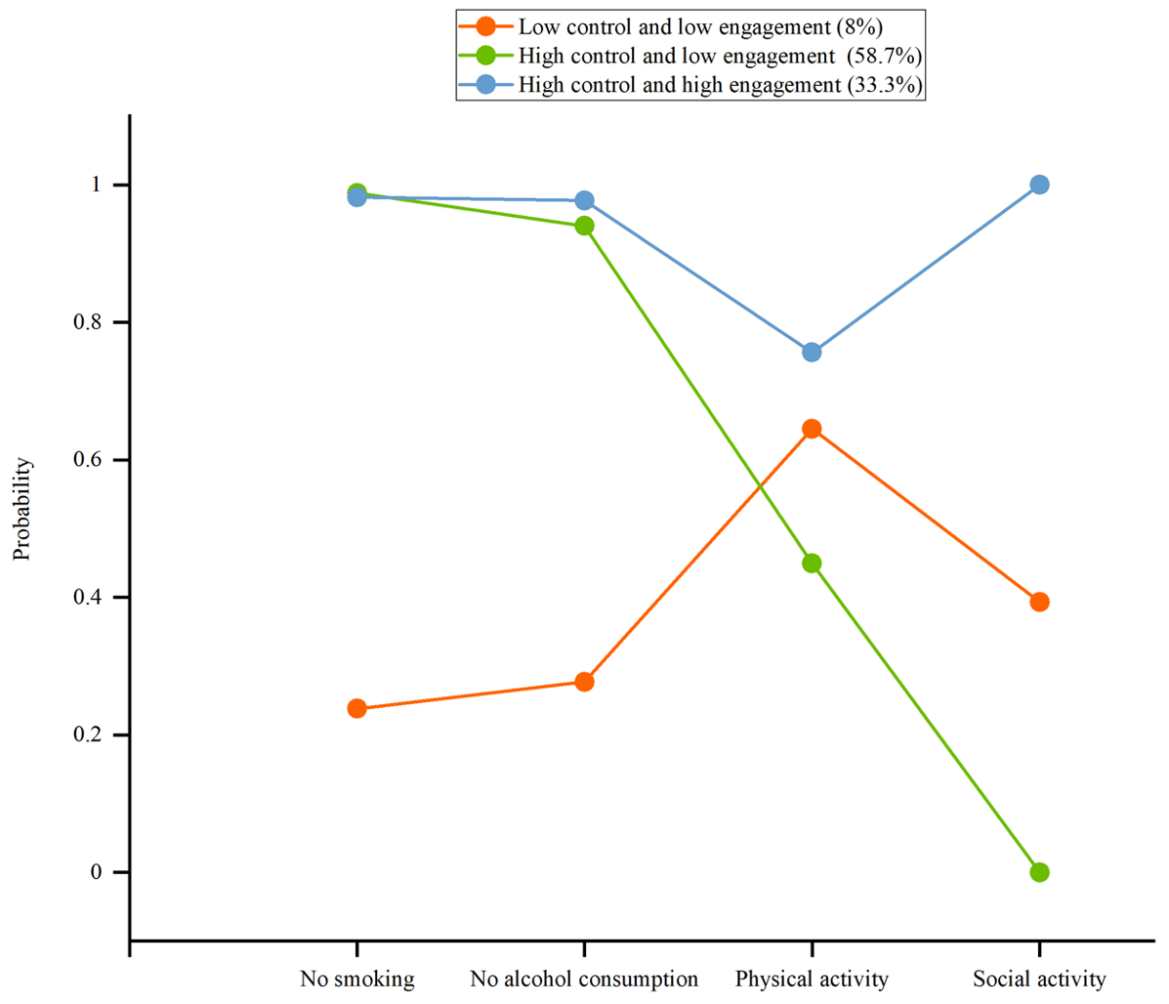
Probability distributions of lifestyle behaviors in the 3 latent classes among community dwelling older adults.

Assigning the name for latent classes was based on a pattern of probability response to items of each latent class and comparison of the probability scale of grouping variables for each latent class. The first class consisted of 8% (165 older adults) and was characterized by low values for all 4 domains. The older adults reported a very low degree of probability in no-smoking and no-drinking domains. Also, older adults reported a low degree of probability in physical activity and social activity domains. Overall, the older adults in this class exhibited lower control levels of smoking and alcohol consumption while demonstrating low engagement in physical and social activities. We called this class “low control-low engagement.” Similarly, class 2 (n=1210, 58.7%) was labeled as “high control-low engagement.” Class 3 (n=685, 33.3%) was labeled as “high control-high engagement.” Figure 1 shows the distribution of lifestyle behavior characteristics across the 3 classes.

**Table 2.** Fit indices for latent class analysis of health lifestyle-related behaviors among community-dwelling older adults.

Model	H0 likelihood value	AIC^a^	BIC^b^	aBIC^c^	Entropy	LMR^d^ *P* value	BLRT^e^ *P* value	Category probability (%)
1	−4009.695	8027.389	8049.911	8037.203	-	-	-	-
2	−3866.229	7750.459	7801.133	7772.539	1.000	<.001	<.001	92.3/7.7
3	−3776.560	7581.120	7659.947	7615.467	0.921	<.001	<.001	8.0/58.7/33.3
4	−3772.757	7583.514	7690.492	7630.128	0.762	.0562	.0505	7.0/2.0/32.2/58.7

a AIC: Akaike information criterion.

b BIC: Bayesian information criterion.

c aBIC: sample size-adjusted Bayesian information criterion.

d LMR: Lo-Mendell-Rubin adjusted likelihood ratio tests.

e BLRT: bootstrap likelihood ratio tests.

### Association Between Lifestyle Behavior Classes and Cognitive Function

In the crude model, both high control-low engagement group (OR 2.026, 95% CI 1.448-2.885) and low control-low engagement group (OR 2.839, 95% CI 1.698-4.677) showed significantly higher risks of cognitive impairment compared to the high control-high engagement group.

In our fully adjusted model, using the high control-high engagement group as the reference, older participants in the high control-low engagement group were more likely to develop cognitive impairment (OR 1.852, 95% CI 1.314-2.655), while those in the low control-low engagement group were also significant (OR 2.905, 95% CI 1.670-5.001). Detailed results are presented in Table 3.

**Table 3.** Association of lifestyle behavior classes and risk of cognitive impairment among community-dwelling older adults.

Variable	Crude			Adjust^b^		
	OR^a^	95% CI	*P* value	OR	95% CI	*P* value
Lifestyle behaviors						
High control-low engagement	2.026	1.448-2.885	<.001	1.852	1.314-2.655	<.001
Low control-low engagement	2.839	1.698-4.677	<.001	2.905	1.670-5.001	<.001
High control-high engagement	1 (Ref.)		8.072	1 (Ref.)		

a OR: odds ratio.

b Adjust model: Logistic regression model adjusted for gender, age, marital status, years of schooling, family economic status, history of coronary heart disease, history of hypertension, history of diabetes, history of hyperlipidemia, BMI, and annual physical examination.

### Subgroup Analysis

**Figure 2.**
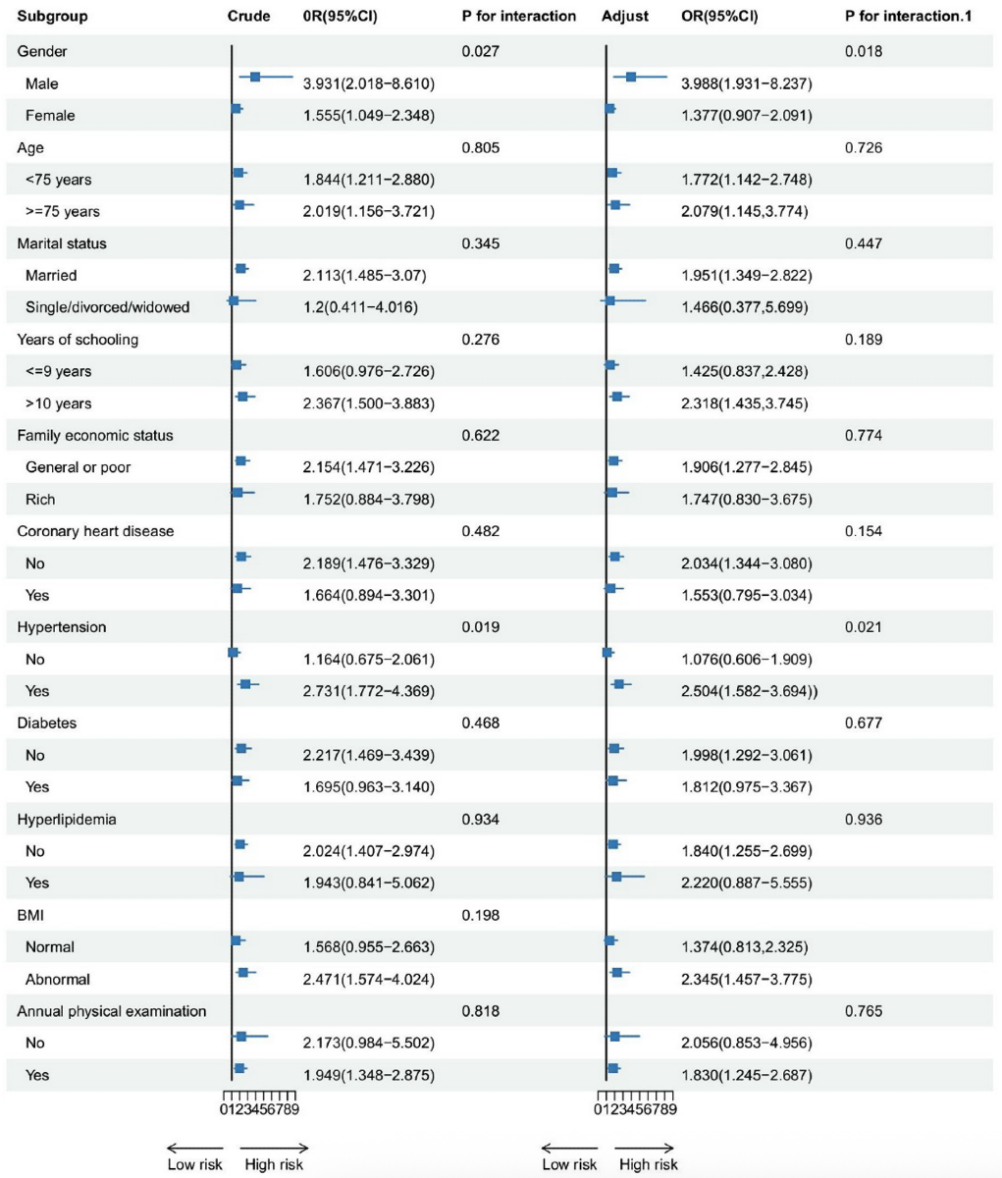
Subgroup analysis of the association between lifestyle classes and risk of cognitive impairment among community-dwelling older adults (high control-low engagement group vs high control-high engagement group). Adjust: model adjusted for gender, age, marital status, years of schooling, family economic status, history of coronary heart disease, history of hypertension, history of diabetes, history of hyperlipidemia, BMI, and annual physical examination. P for interaction refers to the crude model, while P for interaction.1 refers to the adjusted model.

Subgroup analysis showed that there were no statistically significant differences among subgroups in terms of the comparison between low control-low engagement group and high control-high engagement group. Compared to older female adults (OR 1.377, 95% CI 0.907-2.091), high control-low engagement group posed a higher risk of cognitive impairment for older male adults (OR 3.988, 95% CI 1.931‐8.237; *P*=0.018). Compared to older adults without hypertension (OR 1.076, 95%CI 0.606‐1.909), the high control-low engagement group posed a higher risk of cognitive impairment for older adults with hypertension (OR 2.504, 95%CI 1.582‐3.694; *P*=0.021). Detailed results are presented in Figures2  3.

**Figure 3.**
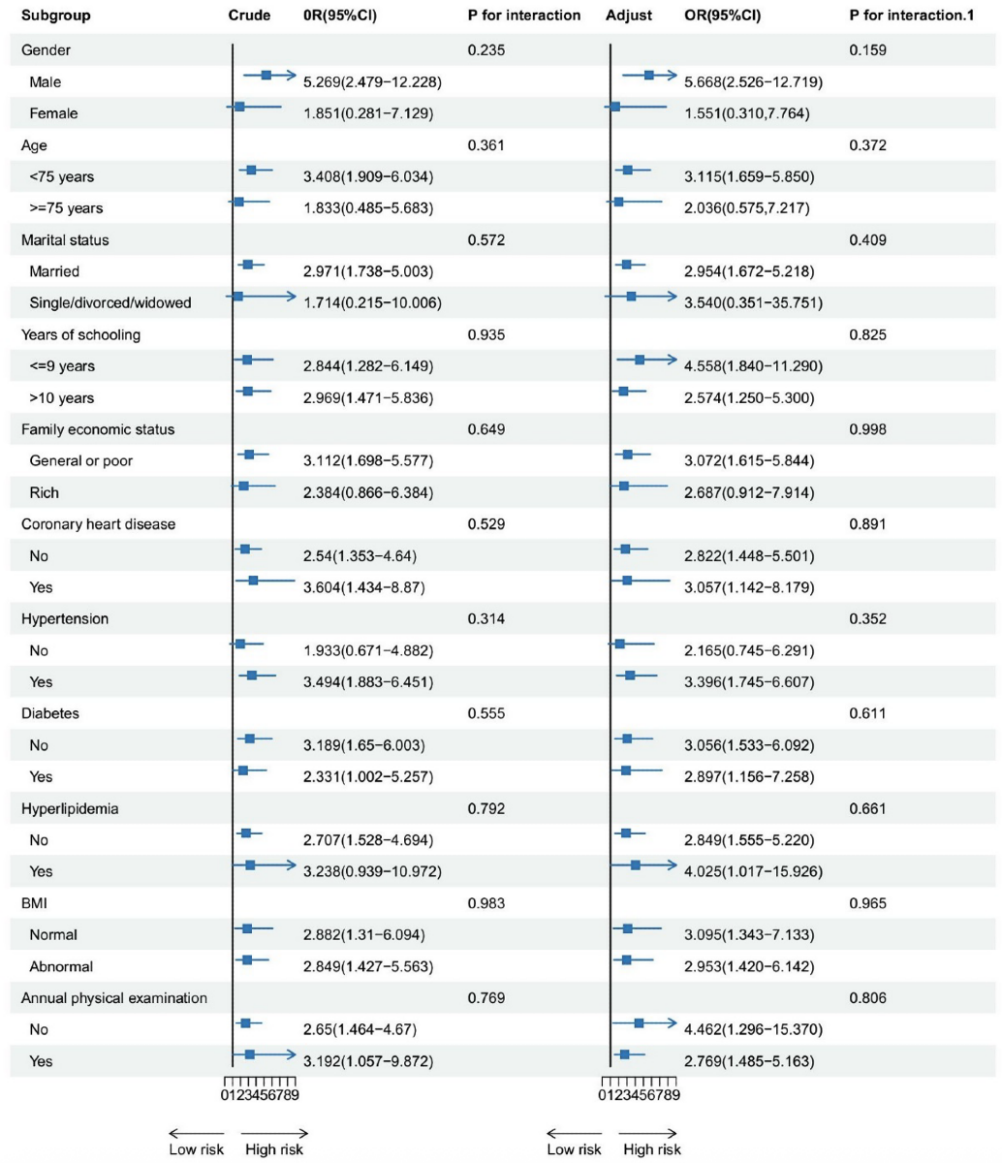
Subgroup analysis of the association between lifestyle classes and risk of cognitive impairment among community-dwelling older adults (low control-low engagement group vs high control-high engagement group). Adjust: model adjusted for gender, age, marital status, years of schooling, family economic status, history of coronary heart disease, history of hypertension, history of diabetes, history of hyperlipidemia, BMI, and annual physical examination. P for interaction refers to the crude model, while P for interaction.1 refers to the adjusted model.

## Discussion

### Principal Findings

Using a large sample of community-dwelling older adults in China, this study used LCA to identify 3 distinct lifestyle behavior classes: high control-low engagement, low control-low engagement, and high control-high engagement group. Individuals in the high control-high engagement group, characterized by non-smoking, moderate alcohol consumption, and frequent physical and social activities, exhibited the lowest risk of cognitive impairment. In contrast, the high control-low engagement and low control-low engagement groups were associated with significantly higher risks (OR 1.852, 95% CI 1.314-2.655 and OR 2.905, 95% CI 1.670-5.001, respectively). These findings underscore the synergistic importance of both avoiding harmful behaviors and actively engaging in health-promoting activities for cognitive health in later life.

Our findings align with existing literature emphasizing the protective effects of multidomain healthy lifestyles on cognitive function [22]. However, this study advances the field by revealing a notable divergence between self-control behaviors (eg, smoking cessation and alcohol moderation) and active engagement behaviors (eg, physical and social activities). While 58.7% of participants exhibited strong self-control but limited engagement, only 33.3% combined both domains optimally. This dichotomy suggests that public health campaigns focusing solely on risk avoidance (eg, anti-smoking policies) may insufficiently address cognitive health unless paired with strategies to promote active engagement. Such insights resonate with Katayama et al [26], who highlighted that holistic lifestyle modifications—rather than isolated behavioral changes—are critical for reversing mild cognitive impairment. Given the challenges of modifying unhealthy behaviors such as smoking and alcohol consumption, encouraging positive behaviors such as increased physical and social activity may be more achievable and impactful. Therefore, health care providers may consider prioritizing interventions that foster physical and social engagement as a foundation, gradually encouraging broader lifestyle improvements over time.

The co-occurrence of smoking and alcohol consumption observed in the low control groups may reflect shared neurobiological pathways, including genetic predispositions to addiction and psychosocial coping mechanisms for stress. Genetically, smoking and alcohol dependence share common genetic markers, increasing the likelihood of co-occurrence [27]. Psychosocially, both behaviors often serve as coping mechanisms for stress and negative emotions, further reinforcing their association [28]. What’s more, the co-occurrence of smoking and alcohol consumption may reflect culturally shaped behavioral synergies in Chinese collectivist contexts. Unlike Western individualistic settings where substance use often occurs in isolation [23], these behaviors in China are deeply embedded in social rituals, for instance, cigarette sharing as a bonding gesture during family gatherings or Chinese liquor consumption as a ceremonial act in community banquets. Conversely, the protective effects observed in the high control-high engagement group may stem from culturally reinforced behavioral synergy. Group-based exercises like morning Tai Chi in parks or evening square dancing serve dual roles as both physical training and social ritual—a phenomenon seldom captured in Western studies [29]. And these activities also offer psychological benefits, such as reducing loneliness, which may further encourage simultaneous participation [30,31]. These findings challenge the direct applicability of Western-derived lifestyle interventions in China. Where previous approaches emphasize individual goal-setting, our results suggest that culturally adapted, group-oriented strategies may be more effective. For instance, redesigning alcohol control campaigns as family-level challenges or framing physical activity as community duty rather than individual choice.

Subgroup analyses further revealed that males in the high control-low engagement group faced a higher risk (OR 3.988, 95% CI 1.931‐8.237) compared to females (OR 1.377, 95% CI 0.907-2.091). This suggests that, under similar conditions of effective control over harmful behaviors (eg, smoking, alcohol consumption), low engagement in physical or social activities presents a greater threat to cognitive health in men. This gender disparity may be attributed to differences in activity intensity, as men are more likely to engage in moderate-to-vigorous physical activities, which confer greater neuroprotective benefits [32]. Additionally, hypertensive individuals in this group exhibited heightened vulnerability, potentially due to the compounding effects of vascular dysfunction and insufficient engagement in cardioprotective activities [33-35]. These findings highlight the need to integrate conventional hypertension management (eg, pharmacotherapy) with behavioral activation strategies aimed at enhancing physical and social engagement.

These findings support the implementation of an LCA-informed stratification framework for managing lifestyle behaviors among China’s aging population. For instance, for the high control-low engagement group, integrate behavioral activation into the National Basic Public Health Service Package. Community nurses could prescribe “Social Exercise Credits”—requiring ≥3 weekly activities (eg, square dancing, Tai Chi clubs), meanwhile, targeting the low control-low engagement group through the Family Doctor Contracted Services and deploying intergenerational health pledges to reduce behavioral risks. Older adults in this group receive monthly home visits jointly conducted by family doctors and their grandchildren. This tiered approach transcends conventional “one-size-fits-all” interventions by precision-targeting latent behavioral profiles while capitalizing on China’s unique familial and policy architectures.

### Limitations

Several limitations warrant consideration. First, the cross-sectional design precludes causal inferences between lifestyle patterns and cognitive outcomes. Second, reliance on self-reported data introduces potential recall bias, particularly for alcohol consumption and social activity metrics. Third, the convenience sampling method and geographic restriction to Beijing may limit generalizability to broader populations. Future studies should use longitudinal designs with objective measures (eg, accelerometry for physical activity, biomarkers for smoking or alcohol use) to validate these associations. Expanding recruitment to diverse regions and incorporating neuroimaging biomarkers could further elucidate the neurovascular mechanisms linking lifestyle behaviors to cognitive decline.

### Conclusions

This study examined the influence of both unhealthy behaviors (smoking and alcohol consumption) and healthy behaviors (frequent engagement in physical and social activities) on cognitive health among older adults. Analytical results demonstrated that individuals in the high control-high engagement group—characterized by non-smoking, moderate alcohol consumption, and frequent physical and social activities—exhibited the lowest risk of cognitive impairment. These findings suggest that public health strategies focused exclusively on risk-reduction interventions (eg, tobacco control policies) may prove insufficient for cognitive protection unless integrated with proactive engagement initiatives, such as the development of community-based physical activity infrastructure and programs to promote social participation among older adults. We propose adopting a dual-pathway intervention model in policy making, simultaneously optimizing risk behaviors management and healthy behaviors promotion mechanisms.

## Abbreviations

LCA: latent class analysis
MMSE: Mini-Mental State Examination

## References

[1] Wimo A, Seeher K, Cataldi R (2023). The worldwide costs of dementia in 2019. Alzheimers Dement.

[2] GBD 2019 Dementia Forecasting Collaborators (2022). Estimation of the global prevalence of dementia in 2019 and forecasted prevalence in 2050: an analysis for the Global Burden of Disease Study 2019. Lancet Public Health.

[3] Ren R, Qi J, Lin S (2022). The China Alzheimer Report 2022. Gen Psychiatr.

[4] Livingston G, Huntley J, Sommerlad A (2020). Dementia prevention, intervention, and care: 2020 report of the Lancet Commission. Lancet.

[5] Deal JA, Power MC, Palta P (2020). Relationship of cigarette smoking and time of quitting with incident dementia and cognitive decline. J Am Geriatr Soc.

[6] Falck RS, Davis JC, Liu-Ambrose T (2017). What is the association between sedentary behaviour and cognitive function? A systematic review. Br J Sports Med.

[7] Kuiper JS, Zuidersma M, Oude Voshaar RC (2015). Social relationships and risk of dementia: a systematic review and meta-analysis of longitudinal cohort studies. Ageing Res Rev.

[8] Lao Y, Hou L, Li J, Hui X, Yan P, Yang K (2021). Association between alcohol intake, mild cognitive impairment and progression to dementia: a dose–response meta-analysis. Aging Clin Exp Res.

[9] Spring B, Moller AC, Coons MJ (2012). Multiple health behaviours: overview and implications. J Public Health (Oxf).

[10] Liu T, Luo H, Tang JY, Wong GH (2020). Does lifestyle matter? Individual lifestyle factors and their additive effects associated with cognitive function in older men and women. Aging Ment Health.

[11] Wang Z, Pang Y, Liu J, Wang J, Xie Z, Huang T (2021). Association of healthy lifestyle with cognitive function among Chinese older adults. Eur J Clin Nutr.

[12] Bloomberg M, Muniz-Terrera G, Brocklebank L, Steptoe A (2024). Healthy lifestyle and cognitive decline in middle-aged and older adults residing in 14 European countries. Nat Commun.

[13] Scotto Rosato N, Baer JC (2012). Latent class analysis: a method for capturing heterogeneity. Soc Work Res.

[14] Mori M, Krumholz HM, Allore HG (2020). Using latent class analysis to identify hidden clinical phenotypes. JAMA.

[15] Zhang J, Zou L, Jiao C (2020). Cognitive benefits of activity engagement among 12,093 adults aged over 65 years. Brain Sci.

[16] Katayama O, Lee S, Bae S (2021). Lifestyle activity patterns related to physical frailty and cognitive impairment in urban community-dwelling older adults in Japan. J Am Med Dir Assoc.

[17] Halloway S, Wagner M, Tangney C (2024). Profiles of lifestyle health behaviors and cognitive decline in older adults. Alzheimers Dement.

[18] Moored KD, Bandeen-Roche K, Snitz BE (2022). Risk of dementia differs across lifestyle engagement subgroups: a latent class and time-to-event analysis in community-dwelling older adults. J Gerontol B Psychol Sci Soc Sci.

[19] Paolillo EW, Saloner R, VandeBunte A, Lee S, Bennett DA, Casaletto KB (2023). Multimodal lifestyle engagement patterns support cognitive stability beyond neuropathological burden. Alzheimers Res Ther.

[20] Hu H, Zhao Y, Guo D (2025). Cognitive function differs across healthy lifestyle behavior profiles: a 10-year population-based prospective cohort study. J Nutr Health Aging.

[21] Norton MC, Dew J, Smith H (2012). Lifestyle behavior pattern is associated with different levels of risk for incident dementia and Alzheimer’s disease: the Cache County study. J Am Geriatr Soc.

[22] Dingle SE, Bowe SJ, Bujtor M (2022). Associations between data-driven lifestyle profiles and cognitive function in the AusDiab study. BMC Public Health.

[23] Zhu W, Li Y (2019). On the differences between Chinese and western cultures from the perspective of cultural dimension theory. Stud Literature Lang.

[24] Li H, Jia J, Yang Z (2016). Mini-Mental State Examination in Elderly Chinese: a population-based normative study. J Alzheimers Dis.

[25] Steinkirchner AB, Zimmermann ME, Donhauser FJ (2022). Self-report of chronic diseases in old-aged individuals: extent of agreement with general practitioner medical records in the German AugUR study. J Epidemiol Community Health.

[26] Katayama O, Lee S, Bae S (2021). Lifestyle changes and outcomes of older adults with mild cognitive impairment: a 4-year longitudinal study. Arch Gerontol Geriatr.

[27] Grucza RA, Bierut LJ (2006). Co-occurring risk factors for alcohol dependence and habitual smoking: update on findings from the Collaborative Study on the Genetics of Alcoholism. Alcohol Res Health.

[28] Ruggles KV, Fang Y, Tate J (2017). What are the patterns between depression, smoking, unhealthy alcohol use, and other substance use among individuals receiving medical care? A longitudinal study of 5479 participants. AIDS Behav.

[29] Luo M, Ding D, Bauman A, Negin J, Phongsavan P (2020). Social engagement pattern, health behaviors and subjective well-being of older adults: an international perspective using WHO-SAGE survey data. BMC Public Health.

[30] Shvedko A, Whittaker AC, Thompson JL, Greig CA (2018). Physical activity interventions for treatment of social isolation, loneliness or low social support in older adults: a systematic review and meta-analysis of randomised controlled trials. Psychol Sport Exerc.

[31] Dahlberg L, Andersson L, Lennartsson C (2018). Long-term predictors of loneliness in old age: results of a 20-year national study. Aging Ment Health.

[32] Williams DM (2008). Increasing fitness is associated with fewer depressive symptoms during successful smoking abstinence among women. Int J Fit.

[33] Ungvari Z, Toth P, Tarantini S (2021). Hypertension-induced cognitive impairment: from pathophysiology to public health. Nat Rev Nephrol.

[34] Lachman S, Boekholdt SM, Luben RN (2018). Impact of physical activity on the risk of cardiovascular disease in middle-aged and older adults: EPIC Norfolk prospective population study. Eur J Prev Cardiol.

[35] Han SH, Tavares JL, Evans M, Saczynski J, Burr JA (2017). Social activities, incident cardiovascular disease, and mortality. J Aging Health.

